# New candidate blood biomarkers potentially associated with white matter hyperintensities progression

**DOI:** 10.1038/s41598-021-93498-w

**Published:** 2021-07-12

**Authors:** Joan Jiménez-Balado, Jesús Pizarro, Iolanda Riba-Llena, Anna Penalba, Júlia Faura, Elena Palà, Joan Montaner, Mar Hernández-Guillamon, Pilar Delgado

**Affiliations:** 1 Neurovascular Research Lab. Vall D’Hebron Research Institute, Universitat Autònoma de Barcelona, Edifici Mediterrània, Planta 1ª, Laboratori 123, Passeig Vall d’Hebron 119-129, 08035 Barcelona, CP Spain; 2 Vall D’Hebron University Hospital, Universitat Autònoma de Barcelona, Dementia Unit, Neurology Service, Barcelona, Spain; 3 Institute de Biomedicine of Seville, IBiS/Hospital Universitario Virgen del Rocío/CSIC/University of Seville & Department of Neurology, Hospital Universitario Virgen Macarena, Seville, Spain

**Keywords:** Neuroscience, Blood-brain barrier, Neuro-vascular interactions

## Abstract

We aimed to discover blood biomarkers associated with longitudinal changes in white matter hyperintensities (WMH). This study was divided into a discovery phase and a replication phase. Subjects in both studies were patients with hypertension, aged 50–70, who underwent two magnetic resonance imaging (MRI) sessions and blood extractions over a 4-year follow-up period. In the discovery phase, we screened 1305 proteins in 12 subjects with WMH progression and in 12 matched control subjects. We found that 41 proteins were differentially expressed: 13 were upregulated and 28 were downregulated. We subsequently selected three biomarkers for replication in baseline and follow-up samples in 80 subjects with WMH progression and in 80 control subjects. The selected protein candidates for the replication were MMP9 (matrix metalloproteinase-9), which was higher in cases, MET (hepatocyte growth factor receptor) and ASAH2 (neutral ceramidase), which were both lower in cases of WMH progression. Baseline biomarker concentrations did not predict WMH progression. In contrast, patients with WMH progression presented a steeper decline in MET over time. Furthermore, cases showed higher MMP9 and lower ASAH2 levels than controls at the follow-up. These results indicate that MMP9, MET, and ASAH2 are potentially associated with the progression of WMH, and could therefore be interesting candidates to validate in future studies.

## Introduction

White matter hyperintensities (WMH) are among the principal hallmarks of cerebral small vessel disease ^1,2^. Radiologically, WMH appear as hyperintense signal lesions in T2 and Fluid Attenuation Inversion Recovery (FLAIR)-weighted sequences ^3^, which, in most cases reflect demyelination and axonal loss as a consequence of sustained exposition to vascular risk factors^4,5^.

Recently, many longitudinal studies have reported substantial rates of progression of WMH during a few years of follow-up^6^, and this progression can be increased in the presence of hypertension and other vascular risk factors^7,8^. WMH spread and conflate on brain parenchyma over time as a subclinical disease, although it is confirmed that this progression significantly impairs cognition^7^ and motor function^9^. However, even though we know the consequences of WMH, the mechanisms implicated in the progression of WMH are poorly understood and the diagnosis is usually incidental.

Importantly, WMH progression has a long covert stage that ultimately results in incident stroke or dementia^10^. Consequently, there is a long therapeutic window in which preventive strategies could attempt to slow or halt this evolution. For this purpose, it is crucial to understand the pathophysiology of WMH, especially if we aim to develop adequate clinical interventions^2^. Additionally, nowadays these changes in WMH can only be detected with the use of serial magnetic resonance imaging (MRI), which complicates the diagnosis of WMH in most settings due to the elevated cost of neuroimaging techniques.

In this context, the study of blood biomarkers might contribute to the achievement of two objectives: increasing the knowledge of the potential causes of WMH, and improving the diagnosis. Previous research has found WMH volume correlated with markers of endothelial dysfunction^11–14^, inflammation^15,16^, kidney function^17^, lipid metabolism^18^, and hemostasis^19^. However, most of these studies had cross-sectional designs, so it was not possible to determine whether these biological processes were involved in the progression of WMH. Moreover, most research has evaluated specific proteins according to a priori hypotheses thus far, but WMH are probably affected by many biological pathways^2^. By contrast, approaches without previous assumptions, such as omics techniques, are rare in the current literature, but these might be more appropriate if we aim to discover new biomarkers for WMH.

This longitudinal population-based study aimed to find blood biomarkers associated with the progression of WMH in patients with hypertension. For that purpose, we first conducted an undriven discovery analysis in the plasma proteome of subjects with and without progression of WMH. Then, three of the proteins that were differentially expressed in the discovery phase of the experiment were validated in a replication phase.

## Methods

### Setting

This study was split into two phases: a discovery phase and a replication phase. Patients from both stages were recruited from the ISSYS cohort (Investigating Silent Strokes in hYpertensive patients, an MRI-based Study)^7,20^. This cohort consists of 976 individuals with hypertension, aged 50–70, who were free of dementia and stroke at the time of enrollment. At the baseline visit (2010–2012), all subjects underwent a brain MRI and blood samples were collected, among other clinical examinations. After 4 years, 350 subjects underwent a follow-up visit (2014–2016) with the same characteristics as at baseline^7^.

This study was in accordance with the declaration of Helsinki and it was approved by the local Ethics Committee at the Vall d’Hebron Institute of Research (VHIR). All patients gave their written, informed consent before inclusion in the baseline and follow-up visits.

### Case and control definitions

Patients underwent a brain MRI at both visits on the same 1.5 Tesla magnetic resonator (Signa HD × 1.5, General Electrics, Waukeska, WI). During both sessions, axial T1, T2, FLAIR, and gradient recall echo-weighted images were acquired. All images had a slice thickness of 5 mm with a 1.5 mm gap, involving 20 axial slices (approximate voxel size of 0.5 × 0.5 × 6.5 mm). Our full neuroimaging protocol has been published previously^20^.

Baseline WMH at periventricular and subcortical areas were quantified according to Fazekas’ scale using both T2 and FLAIR sequences, in which WMH appear as hyperintense lesions^21^. Presence of extensive WMH at baseline was defined as a score in Fazekas’ scale greater than or equal to 2 at the periventricular and/or subcortical areas.

WMH longitudinal changes were measured according to the Rotterdam Progression Scale (RPS) using FLAIR and T2 images. Briefly, this scale quantifies the qualitative change in WMH by comparing baseline and follow-up MRIs in a side-by-side manner^4^. Progression, stability, or recession of WMH is evaluated in different anatomical areas at the subcortical and periventricular locations, and subjects are given a score ranging from − 14 to 14. This assessment was conducted by two independent readers blinded to clinical data and time of the MRI. We evaluated the inter- and intra-reliability of both readers using the intraclass correlation coefficient, obtaining adequate reliability for both subcortical and periventricular WMH assessments (> 0.70 in both cases)^7^.

In both discovery and replication experiments, cases with progression of WMH were considered as those subjects showing a total RPS score greater than or equal to 3^22^. Otherwise, individuals were considered controls.

### Subjects in the discovery and replication studies:

**Figure 1:**
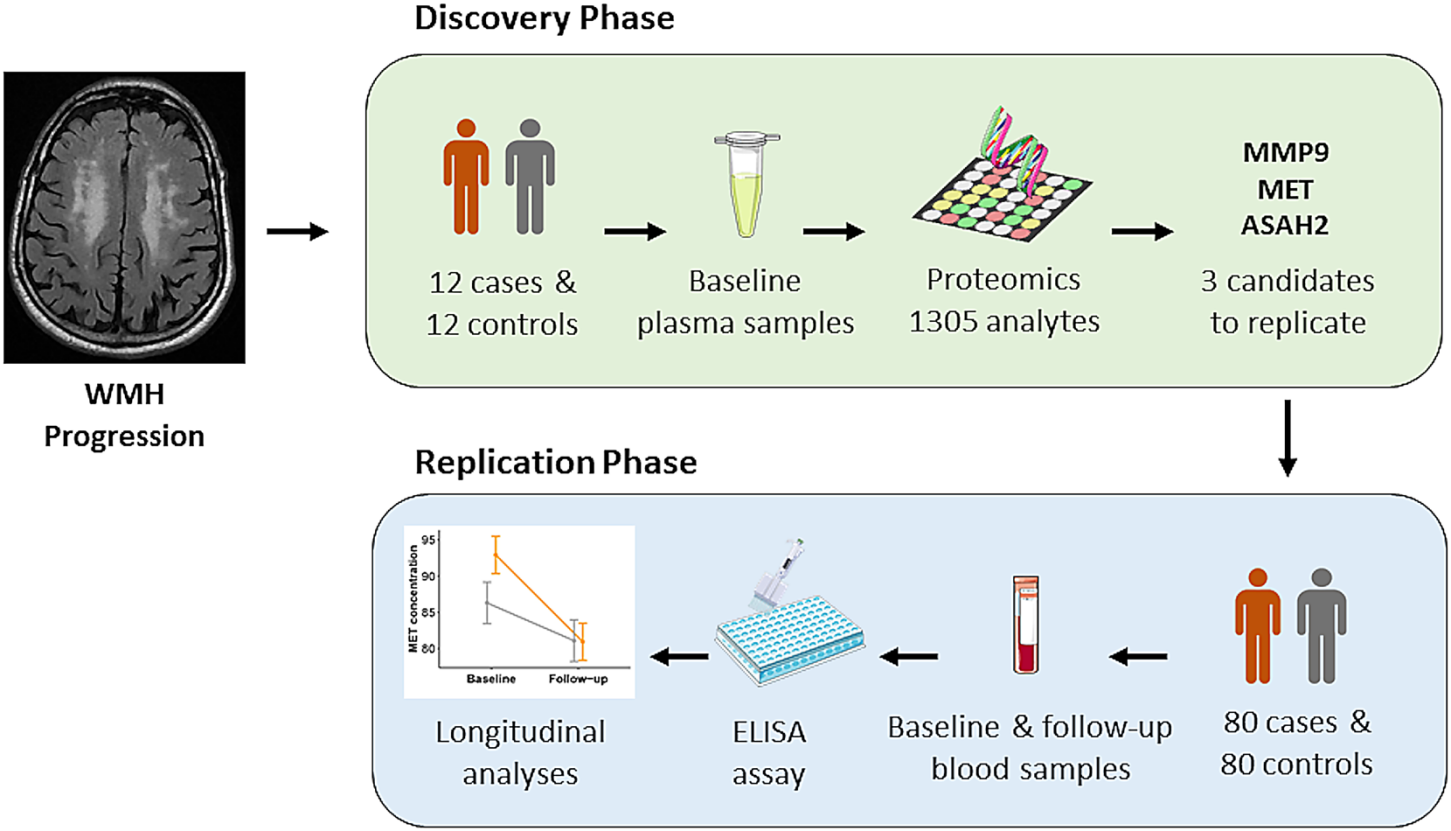
Study design and number of patients in each phase. Discovery and replication phases were conducted within the ISSYS cohort (patients with hypertension, aged 50–70, and without dementia and stroke at the baseline visit). In both phases of the study, progression of WMH was defined as a total RPS score greater than or equal to 3. In the discovery phase, cases and controls were matched by age, sex, and baseline WMH burden, while in the replication phase, cases and controls were matched by age and sex. Parts of this figure were supported by Servier Medical Art (https://smart.servier.com/) with permission under the Creative Commons Attribution 3.0 Unported License. The figure was constructed using Microsoft Office Powerpoint 365 (version 2013, https://www.microsoft.com/en-us/microsoft-365/powerpoint), GNU image manipulation software (GIMP, version 2.10.22, https://www.gimp.org/) and ‘ggplot’ library (version 3.3.2, https://ggplot2.tidyverse.org/) included in R software. (R version 3.6.3, 2020-02-29; 2020 The R Foundation for Statistical Computing, https://www.r-project.org/). ASAH2, neutral ceramidase; ISSYS, Investigating Silent Strokes in hYpertensives Study; MET, hepatocyte growth factor receptor; MMP9, matrix metalloproteinase-9; RPS, Rotterdam progression scale; WMH, white matter hyperintensities.

In the discovery phase, we selected 12 cases (subjects with progression of WMH) and 12 controls, matched by age, sex, and baseline WMH burden, to conduct a proteomic experiment. The selected markers from the discovery phase were replicated in a sample consisting of 80 cases and 80 controls, paired by age and sex. Among these subjects, 11 out of 12 pairs of subjects from the discovery phase were also included in the replication phase to validate the SOMAscan technique with an orthogonal method (Enzyme-Linked Immunosorbent Assay [ELISA]). Figure 1 shows a graphical representation of the study design.

### Sample processing

Blood samples were drawn from each participant during both visits. Serum and plasma were obtained after 15 min centrifugation at 3500 rpm and frozen at − 80 ºC for further analysis.

#### Discovery phase

The discovery study was performed by means of SOMAscan (SomaLogic Inc., Boulder, CO, USA), which is an aptamer-based proteomic assay^23^. In this assay, 1305 protein analytes were measured in baseline plasma samples (100 µL each) from 12 cases and 12 controls. This approach uses SOMAmers, which are modified nucleic acid aptamers resistant to DNA nucleases, to convert a protein signal to a nucleotide signal, which is quantified using relative fluorescence on microarrays. Units represent relative fluorescence units (RFU). All samples passed quality controls. Three different dilutions were analyzed to separate groups of high, medium, and low abundance proteins. Normalization and calibration procedures following the SOMAscan protocol were conducted in order to remove systematic error in raw data^24^.

#### Replication phase

Protein candidates selected during the discovery phase (matrix metalloproteinase-9 [MMP9]; hepatocyte growth factor receptor [MET]; neutral ceramidase [ASAH2]) were measured in baseline and follow-up samples of 80 cases and 80 controls by means of quantitative Enzyme-Linked Immunosorbent Assay (ELISA). Specifically, MMP9 was measured by human MMP9 ELISA assay (GE Healthcare, Chicago, IL) in plasma samples. MET and ASAH2 were measured using Human MET/ASAH2 ELISA kits (Cloud-Clone Corp, Houston, TX) in serum samples. Assays were conducted according to the manufacturers’ instructions. Moreover, the sample concentration of each molecule was measured in duplicate, and the mean of the two values was used in data analysis. Those samples having a coefficient of variation (CV) greater than 20% between the two concentration values were excluded from the analysis. According to this criteria, 13 samples were excluded for MET (7 were baseline samples and 6 follow-up samples), and 12 were excluded for ASAH2 (all baseline samples). Plate-to-plate variation was determined by including a commercial sample pool in each plate and calculating the CV between plates. Concentration was expressed in ng/mL.

### Covariables

Information about demographic and vascular risk factors was collected at the baseline and follow-up visits. Mean systolic (SBP) and diastolic blood pressures were calculated as the mean of two out of three measurements after 5 min of rest at both visits. Cholesterol and high-density lipoprotein (HDL)-cholesterol were measured automatically via a clinical analyzer (Olympus AU 2007).

### Statistics

All analyses were conducted using R software (R version 3.6.3, 2020-02-29; 2020 The R Foundation for Statistical Computing, https://www.r-project.org/).

Characteristics of individuals with progression of WMH and individuals without progression were compared by means of *t*-test, U Mann–Whitney, or χ^2^, depending on the type and distribution of the variable. To analyze the change in biomarker concentrations between visits during the replication phase of the study, we used paired samples *t*-tests.

#### Discovery phase

For the discovery phase of the study, proteomic data was analyzed using the “limma” package (Bioconductor, version 3.34.9)^25^. This package allows data from omics experiments to be analyzed in an efficient way. First, proteins from the SOMAscan array (n = 1305) were log-transformed, since they presented a skewed distribution. Then, linear models were constructed for each feature, introducing the phenotype (cases versus controls) as the predictor variable. The log fold change (LogFC) indicates whether a protein is up- or downregulated in cases. *P*-values were adjusted according to false discovery rate (FDR) correction. Finally, we conducted a ranked enrichment analysis for pathways (Reactome) and for molecular functions and biological processes (Gene Ontologies [GO]), considering the full SOMAscan data arranged by *t*-statistic, following the directions of a previously published protocol^26^. This analysis was conducted by means of GSEA^27^ software (Gene Set Enrichment Analysis, 2004–2020; Broad Institute, Inc., Massachusetts Institute of Technology, and Regents of the University of California, https://www.gsea-msigdb.org/gsea/index.jsp) and results were visualized via Cytoscape (version 3.8.2, 2001–2018 Cytoscape Consortium, https://cytoscape.org/)^26,28^. Significant pathways at the exploratory level were considered at *q*-value < 0.25^26^.

#### Replication phase

For the replication phase, we first used mixed models to compare the differences in biomarker concentrations between cases and controls, and within visits. Models were constructed using the biomarker concentration as the dependent variable, and group (cases and controls), time, and time × group interaction were the independent variables of interest. This interaction indicated whether longitudinal changes in biomarker concentrations were the same or different between cases and controls.

Then, these models were adjusted for potential confounders. Covariables were selected according to whether they were associated with our biomarker candidates or with the progression of WMH (*p*-value cutoff < 0.1). We adjusted for sex, baseline age, change in SBP, visit (baseline vs follow-up), and baseline burden of WMH. Multivariate results were visualized by extracting the marginal means of each group at each time.

In all models, random intercepts were considered for subject identification. Random slopes were not evaluated since we determined biomarker levels at only two time points.

### Data sharing

We will share anonymized data upon the request of qualified researchers.

## Results

### Discovery phase

#### Differentially expressed proteins

**Figure 2:**
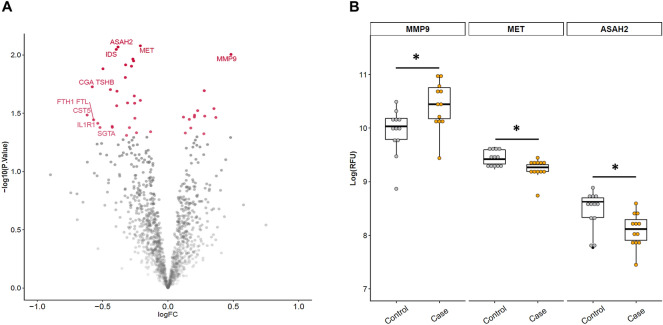
Discovery phase results. (**A**) Volcano plot of the discovery study. The y-axis represents − log10 *p-*value, and the x-axis represents the log2 fold change. Values greater or less than 0 indicate upregulated and downregulated genes, respectively. Color and opacity indicate whether features had a significant nominal *p*-value (red and opaque) or not significant (grey and transparent). Labeled features are those with a nominal *p*-value < 0.05 and |logFC|> 0.5 or *p*-value < 0.01. (**B**) Boxplots of RFU levels of the three selected protein candidates replicated from the discovery study (n = 24). The y-axis represents log(RFU) and the x-axis represents the group (controls, grey; cases, orange). *: *P*-value < 0.01. ASAH2, neutral ceramidase; MET, hepatocyte growth factor receptor; MMP9, matrix metalloproteinase-9; RFU, relative fluorescence units.

Median age of the discovery sample was 64 years (range of 62–67, n = 24). Cases and controls did not differ in any of the potential confounders (subclinical cerebrovascular lesions, sex, and age), and we observed that only individuals with progression presented an increase in SBP from baseline to follow-up visit (see Supplementary Table 1). Among the markers we screened, 41 were associated with the progression of WMH at a nominal *p*-value of < 0.05. Of these 41 proteins, 13 were upregulated and 28 were downregulated. Among them, 4 markers showed a nominal *p*-value of < 0.01: MET, ASAH-2, iduronate 2-sulfatase (IDS), and MMP9. Specifically, MET, ASAH-2, and IDS were downregulated, and MMP9 was upregulated. Differences in protein expression levels did not remain significant after FDR correction. Figure 2-A shows the volcano plot of this analysis. In Supplementary Table 2, we show all the proteins in the discovery study that were differentially expressed at a nominal *p*-value of < 0.05.

After reviewing the literature, MMP9, MET, and ASAH-2 were selected as biomarkers for the replication phase of the study, because these candidates have been previously connected to cerebrovascular or other neurological diseases^11,29–32^. In Fig. 2-B, we show the boxplots representing differences in baseline RFU between cases and controls. LogFCs of MMP9, MET, and ASAH2 were 0.48, − 0.21, and − 0.38, respectively.

#### Differentially expressed pathways

**Figure 3:**
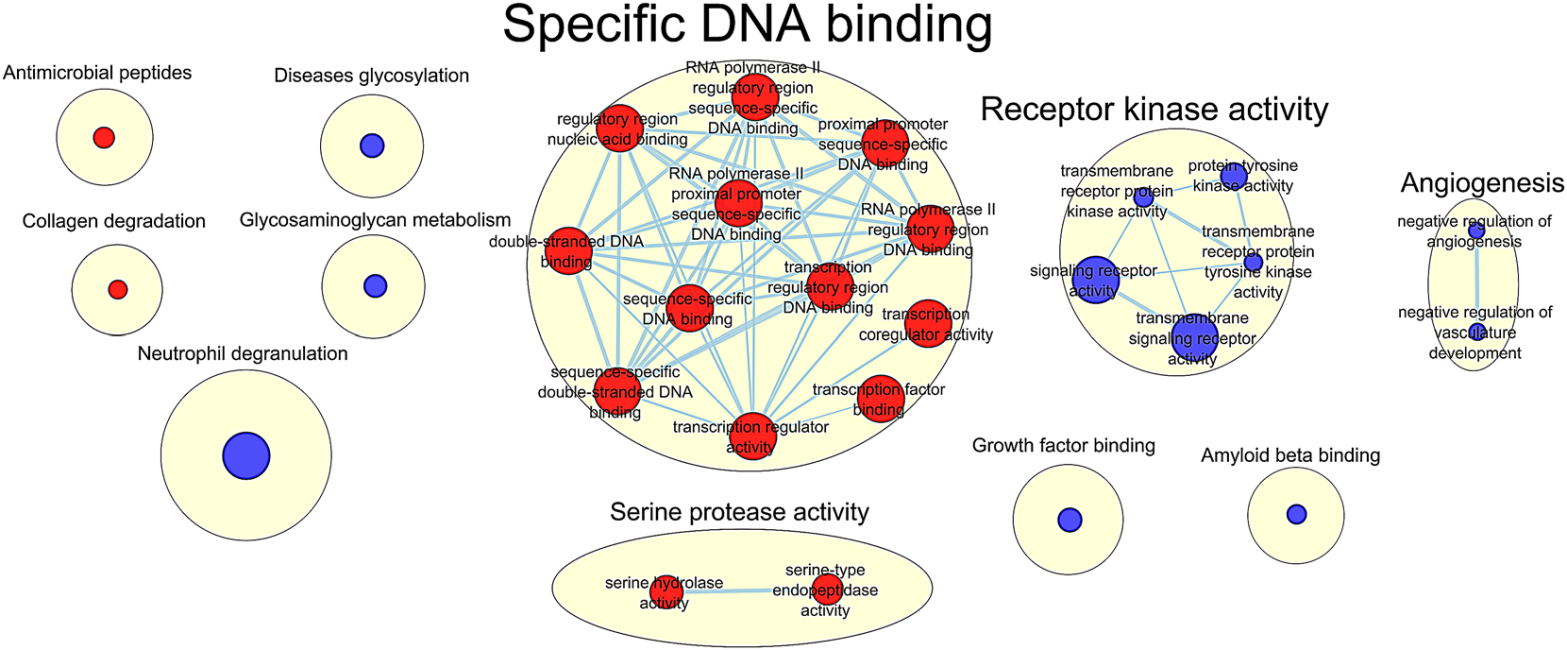
Enriched Reactome and Gene Ontologies (biological processes and molecular functions) pathways in the proteomic data. 5 Reactome pathways, 21 GO molecular functions, and 2 GO biological processes were significantly enriched (q-value < 0.25). Red circles: upregulated processes in patients with progression of WMH; blue circles: downregulated processes. 5 pathways were significantly enriched (at q-value < 0.25) regarding the Reactome, 21 considering GO molecular function and 2 considering GO biological processes. This figure was constructed with Cytoscape software (version 3.8.2, 2001–2018 Cytoscape Consortium, https://cytoscape.org/). Annotations correspond to the most frequent words in each cluster.

Regarding GSEA (Gene Set Enrichment Analysis analysis), we found significant enrichment (*q*-value < 0.25) of proteins related to 2 GO biological process pathways, 21 GO molecular functions, and 5 Reactome pathways (the complete list of these gene sets can be found in Supplementary Table 3). As represented in Fig. 3, these pathways included ubiquitous processes, such as specific DNA binding and receptor kinase activity. Other interesting pathways were neutrophil degranulation, amyloid beta binding, angiogenesis, and collagen degradation, among other functions.

Among the enriched pathways, 4 sets included MMP9 and 5 included MET (Supplementary Table 4). None of the enriched gene sets included ASAH2. MMP9 was involved in processes such as collagen degradation and neutrophil degranulation, while MET was principally involved in receptor kinase activity.

### Replication phase

#### Descriptive analysis

In Table 1, we show the principal characteristics of the replication sample, split by cases and controls (n = 160). Median age of the sample was 65 years (range 62–68) and 70 subjects (43.8%) were female. Cases showed a greater increase in SBP within visits, as well as a higher burden of baseline WMH, than controls. In this phase, MMP9, MET, and ASAH were measured by ELISA, and inter-plate CVs were under 15% in all cases.

**Table 1:** Characteristics of the replication phase sample by cases and controls.

	Controls (n = 80)	Cases (n = 80)	*P*-value
Baseline age, years	65 (62;68)	65 (62;67)	0.876
Sex	45 (56.3)	45 (56.3)	1
Time between visits, years	4.0 (3.8;4.1)	4.0 (3.8;4.3)	0.810
**Vascular risk factors**			
Diabetes mellitus	16 (20.0)	14 (17.5)	0.840
Active smoker	8 (10.0)	10 (12.5)	0.800
SBP, mmHg	144.9 (15.31)	141.1 (18.4)	0.149
**ΔSBP, mmHg**	**1.3 (14.7)**	**8.4 (19.7)**	**0.011**
DBP, mmHg	78.2 (9.4)	78.2 (9.4)	0.993
ΔDBP, mmHg	− 3.7 (8.9)	− 1.3 (10.1)	0.120
Cholesterol, mg/dL	216.4 (44.6)	214.3 (43.5)	0.766
ΔCholesterol, mg/dL	− 2.1 (41.0)	− 8.1 (40.6)	0.362
HDL cholesterol, mg/dL	46.8 (38.7;58.6)	47.5 (40.5;57.8)	0.644
ΔHDL cholesterol, mg/dL	3.7 (− 0.3;10.3)	3.6 (− 1.8;9.5)	0.656
**Baseline cerebral small vessel disease**			
Extensive PVH	12 (15.0)	23 (28.8)	0.06
**Extensive DWMH**	**4 (5.0)**	**27 (33.8)**	** < 0.01**
Silent brain infarct	12 (15.0)	22 (27.5)	0.08

Values represent mean (standard deviation [SD]), median (interquartile range), or number (%). Bolded variables are those which show significant differences between cases and controls (*p*-value < 0.05).

DBP, diastolic blood pressure; DWMH, deep white matter hyperintensities; HDL, high-density lipoprotein; PVH, periventricular hyperintensities; SBP, systolic blood pressure.

We first compared the baseline biomarker concentration between individuals with extensive and non-extensive baseline WMH (Fazekas’ scale ≥ 2). Individuals with baseline extensive WMH presented reduced MET levels compared to individuals with non-extensive burden (mean [standard deviation], x̄_extensive_ = 85.2 ng/mL [20.3] vs x̄_non-extensive_ = 95.4 ng/mL [22.1], *p-*value = 0.007). By contrast, there were no differences for MMP9 or ASAH (*p*-value > 0.05).

#### Validation of SOMAscan technique

Among the subjects in the replication phase, 22 individuals were also present in the discovery study. In this subsample, cases presented a higher baseline MMP9 concentration as compared to controls (mean [standard deviation], x̄_cases_ = 37.4 ng/mL [10.8] vs x̄_controls_ = 27.3 ng/mL [11.6], *p*-value = 0.05). However, this was not observed for MET and ASAH2 (Supplementary Fig. 1). This suggests that the SOMAscan technique was validated by ELISA for MMP9, but not for MET or ASAH2.

#### Longitudinal analysis

In Supplementary Fig. 2-A, we compare the mean biomarker concentration from baseline to follow-up visits in the replication study. We found that MMP9 and MET significantly decreased within visits, while ASAH2 increased (all *p*-values < 0.001). In Supplementary Fig. 2-B, these results have been split by group (cases and controls). We found that biomarker levels changed in both cases and controls following the same trend that was observed in the whole sample. However, we found a significant interaction between group and visit for MET (β [95% CI, confidence interval] − 7.13 [− 12.3 to − 2.0]; *p*-value = 0.01), which suggested that cases presented a more pronounced decline in MET within visits compared to controls.

**Figure 4:**
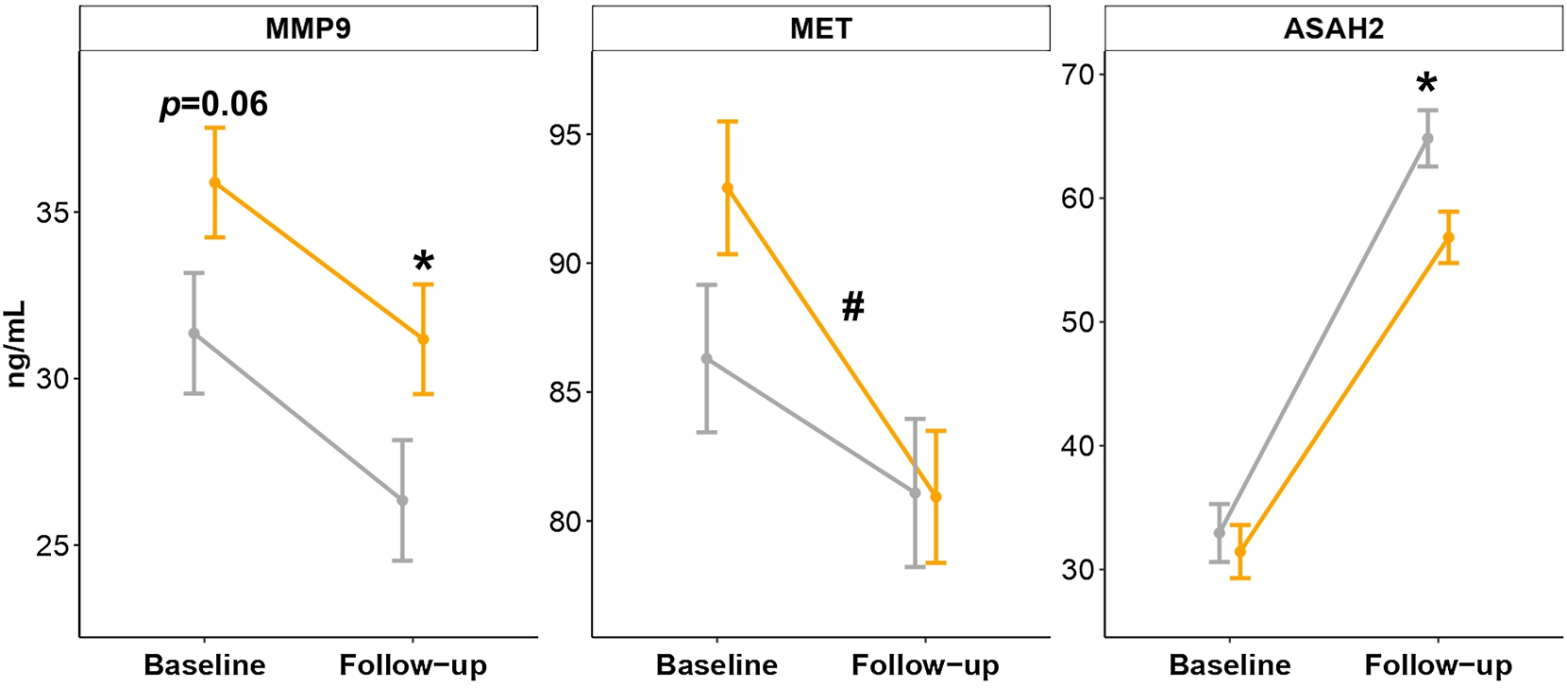
Biomarker concentrations in the replication phase (n = 160). Lines represent adjusted means from baseline to the follow-up visit in cases (black) and controls (grey). Error bars represent the standard error of the adjusted mean. These adjusted means have been extracted from mixed models and were corrected for baseline age, sex, visit, change in systolic blood pressure, and baseline WMH. *: *p*-value < 0.05 between groups at the baseline visit or at the follow-up visit. #: group and visit interaction *p*-value < 0.05. This interaction term represents the difference in the rate of changes between groups in biomarker concentrations.

In Fig. 4, we present these results adjusted for sex, baseline age, change in SBP, visit (baseline vs follow-up), and baseline burden of WMH. Plotted values represent the marginal means of these multivariate models for group and visit interaction, which remained statistically significant for MET (β [95% CI] − 6.8 [− 1.7 to − 11.9]. *p*-value = 0.01), confirming that patients with progression of WMH had a steeper decline in this marker over time. On the other hand, compared to controls, cases showed a higher concentration of MMP9 at the follow-up visit (adjusted mean [standard error], x̄_cases_ = 31.2 ng/mL [1.7] vs x̄_controls_ = 26.3 ng/mL [1.8]; *p*-value = 0.05) and a lower concentration of ASAH2 (x̄_cases_ = 56.8 ng/mL [2.1] vs x̄_controls_ = 64.8 ng/mL [2.3]; *p*-value = 0.01).

Contrary to our expectations, subjects with progression of WMH did not differ in the baseline concentrations of any of the studied proteins, indicating that no biomarker could predict the progression of WMH in the replication study. We found only a borderline association for baseline MMP9 concentration (*p*-value = 0.06, Fig. 4).

As cases presented a higher baseline WMH burden than controls in the replication sample (Table 1), we ran a sensitivity analysis in 55 out of 80 pairs of subjects showing no differences in baseline WMH according to Fazekas’ scale (see Supplementary Table 5 for a descriptive analysis of this sub-sample). Interestingly, we observed the same associations as presented above, indicating that the relationship between these proteins and progression of WMH was not confounded by baseline WMH (Supplementary Fig. 3).

## Discussion

In this study, we screened 1305 proteins from the plasma of patients with progression of WMH. We then selected three biomarker candidates (MMP9, MET, and ASAH2) to replicate in a second phase of the experiment, and studied whether changes in biomarker concentrations over time were observed in parallel to WMH progression. Contrary to our expectations, no biomarker predicted the progression of WMH, although there was a trend for MMP9 (*p*-value = 0.06), making this protein an interesting target for future validation studies. Still, patients with progression of WMH showed a steeper decline in MET concentration within visits and had differentially expressed MMP9 and ASAH2 levels at the follow-up visit.

MET is a tyrosine kinase receptor of the hepatocyte growth factor (HGF), which is expressed in many tissues. Indeed, tyrosine kinase receptor activity was downregulated in cases in the GSEA analysis. In the brain, HGF/MET activation is involved in synaptic plasticity, survival, proliferation, and angiogenesis, among other processes, via the phosphatidylinositol 3-kinase-Akt/Protein Kinase B (PI3K/AKT) pathway^29^. Higher serum and cerebrospinal fluid (CSF)-HGF concentrations have been previously associated with increased WMH burden in several cross-sectional studies in patients with Alzheimer’s disease and subjective memory complaints^31,33^. Moreover, increased serum HGF levels indicated higher stroke risk in older adults without previous symptomatic cardiovascular disease^34^. In our study, we observed that a decrease in MET paralleled an increase in WMH, and individuals with baseline extensive WMH had lower MET levels than individuals without extensive WMH. One possible explanation might be that the decrease in MET within visits reflects the downregulated activity of neuroprotective processes (downregulated tyrosine kinase pathways) and angiogenesis, which might be explained by a lower HGF/MET binding. Of note in the pathway analysis, angiogenesis was downregulated in cases, although MET did not belong to this pathway. Hence, another possibility might be that MET concentration is returning to “control” levels, and the increase observed at the baseline visit reflects a response to a harmful stimulus. In light of these results, we propose that future studies should measure both MET and HGF to understand how these biomarkers are interacting with each other in the development of WMH.

Patients with progression of WMH showed a higher concentration of MMP9 at the follow-up visit and exhibited a trend towards increased baseline levels (*p*-value = 0.06). MMP9 and other metalloproteinases (MMPs) are responsible for collagen IV degradation, leading to blood–brain barrier (BBB) disruption^35^. Interestingly, collagen degradation was upregulated in cases, as were other biological processes, such as neutrophil degranulation, that might appear in response to BBB breakdown. As summarized in several review articles, there have been several attempts to measure MMPs in blood samples of individuals with WMH, which provided some significant results, although with small effect sizes, complicating the translation of these findings into clinical practice^11,35,36^. By contrast, studies measuring MMP9 in CSF reported more consistent and stronger results, such as elevated MMP9 in patients presenting vascular cognitive impairment^37,38^. To our knowledge, only one other group has studied whether baseline MMP9 levels predict the progression of WMH, and they found a significant association with a moderate effect size^39^. In our study, however, differences were only observed at the follow-up visit, indicating that MMP9 levels did not predict the progression of WMH. Nonetheless, we suggest that, given the borderline association observed at the baseline visit, MMP9 is a good candidate to validate in independent cohorts, along with related proteins, such as tissue inhibitor of metalloproteinases (TIMPs) or other MMPs.

On the other hand, patients showing an increase in WMH presented lower ASAH2 levels at the follow-up visit. ASAH2 is involved in sphingolipid metabolism hydrolyzing ceramides, preventing its accumulation^40^. Ceramide accumulation has been shown to be associated with incident cardiovascular disease and cognitive impairment^32,41^. Similarly, deregulation of sphingolipid metabolism has been described in other neurological pathologies, such as Alzheimer’s disease^42^. However, to our knowledge, no study has described an association between ASAH2 and the progression of WMH.

Importantly, we included 11 out of the 12 pairs of subjects from the discovery phase in the replication phase of this study. In this subset, we could only validate the SOMAscan technique for MMP9. These results align with another study, which had overlapping data from 63 markers measured using both the SOMAscan platform and conventional multiplex immunoassays. In that study, MMP9 presented a moderate reliability between techniques (0.46–0.63), although 20% of their analytes presented lack of correlation (r < 0.3)^43^. There are two main factors that could be involved in the disparity between these techniques. First, the most likely cause might be that different binding reagents interact with different epitopes of a specific analyte. Consequently, binding reagents might present further disparities in results due to protein-to-protein interactions in a complex matrix, depending on whether these interactions involve one of the reagent’s binding sites. Second, cross-reactivity and negative cooperative binding could also lead to diminished specificity and sensitivity in one or both techniques^44^. Hence, further discovery studies aiming to find biomarker candidates via an aptamer-based strategy should include independent technique validations.

Altogether, we have presented several proteins and biological processes that relate to WMH changes over time. It is important to consider WMH as a complex disease in which several causes may be involved in its pathogenesis. Hence, one biomarker may be not enough to predict the evolution of WMH and to combine the information of several markers in a panel may be of interest. In this regard, omics techniques, in combination with longitudinal studies, may help to develop new diagnostic tools and a better understanding of WMH pathophysiology. Moreover, previous results from our group indicate that a marked progression of WMH according to RPS, as defined in this study, increases the risk of incident mild cognitive impairment^7^. Therefore, from a clinical point of view, finding new diagnostic tools assessing the risk of WMH progression may help to detect those patients who, to prevent cognitive impairment, need more strict medical follow-ups to monitor vascular risk factors.

This study has several strengths and limitations. One limitation is the sample size, which restricted the statistical power to correct for multiple comparisons in both the discovery and replication phases. Furthermore, we only analyzed a small portion of the full proteome, and this likely conditioned our findings, especially regarding the pathway analysis. Mass spectroscopy studies should be considered in future research. Moreover, in the replication phase, SOMAscan results were not validated by ELISA technique for MET and ASAH2. Therefore, our results should be interpreted with care and should be validated in larger studies. Considering that the concentration of these biomarkers presented a statistically significant change within time in our study, suggesting that their concentration may be influenced by aging or other unknown factors, future studies should be longitudinal. Finally, in the replication phase, we only evaluated our biomarker candidates at two points in time and, thus, we can only model the changes in a linear fashion. However, the temporal profile of these proteins might present nonlinear shapes, and future studies should account for this.

However, our study may be considered as a hypotheses-generating experiment. In our analysis, we propose several mechanisms and proteins that may be implicated in the progression of WMH. We conducted a proteomic discovery study followed by a replication study, which brings reliability to our results. Progression of WMH was qualitatively defined by a previous cutoff that may be easily exported to future research with multicenter studies^45^.

## Conclusions

In conclusion, in this study we ran a discovery study in the plasma proteome of 12 patients showing WMH progression and 12 controls, finding 41 proteins which were differentially expressed in cases (28 were downregulated and 13 were upregulated). In the replication phase, we aimed to validate 3 proteins (MMP9, MET and ASAH2) in 80 cases and 80 controls. Thereby, we observed that decline in MET serum concentration paralleled the increase in WMH. Moreover, MMP9 and ASAH2 were differentially expressed in cases at the follow-up visit. Although none of these markers predicted the progression of WMH and discovery phase results were only validated for MMP9, patients with progression of WHM showed specific temporal profiles for these markers. Consequently, we propose that these proteins might be good candidates to validate in future studies having larger sample sizes.

## Supplementary Information

Supplementary Information.
