# Supplementary material for: New candidate blood biomarkers potentially associated with white matter hyperintensities progression
Source: Sci Rep. 2021 Jul 12;11:14324. doi: 10.1038/s41598-021-93498-w (PMC8275657; doi:10.1038/s41598-021-93498-w)
Supplement: Supplementary file 1 — Supplementary Information. [file 41598_2021_93498_MOESM1_ESM.docx]

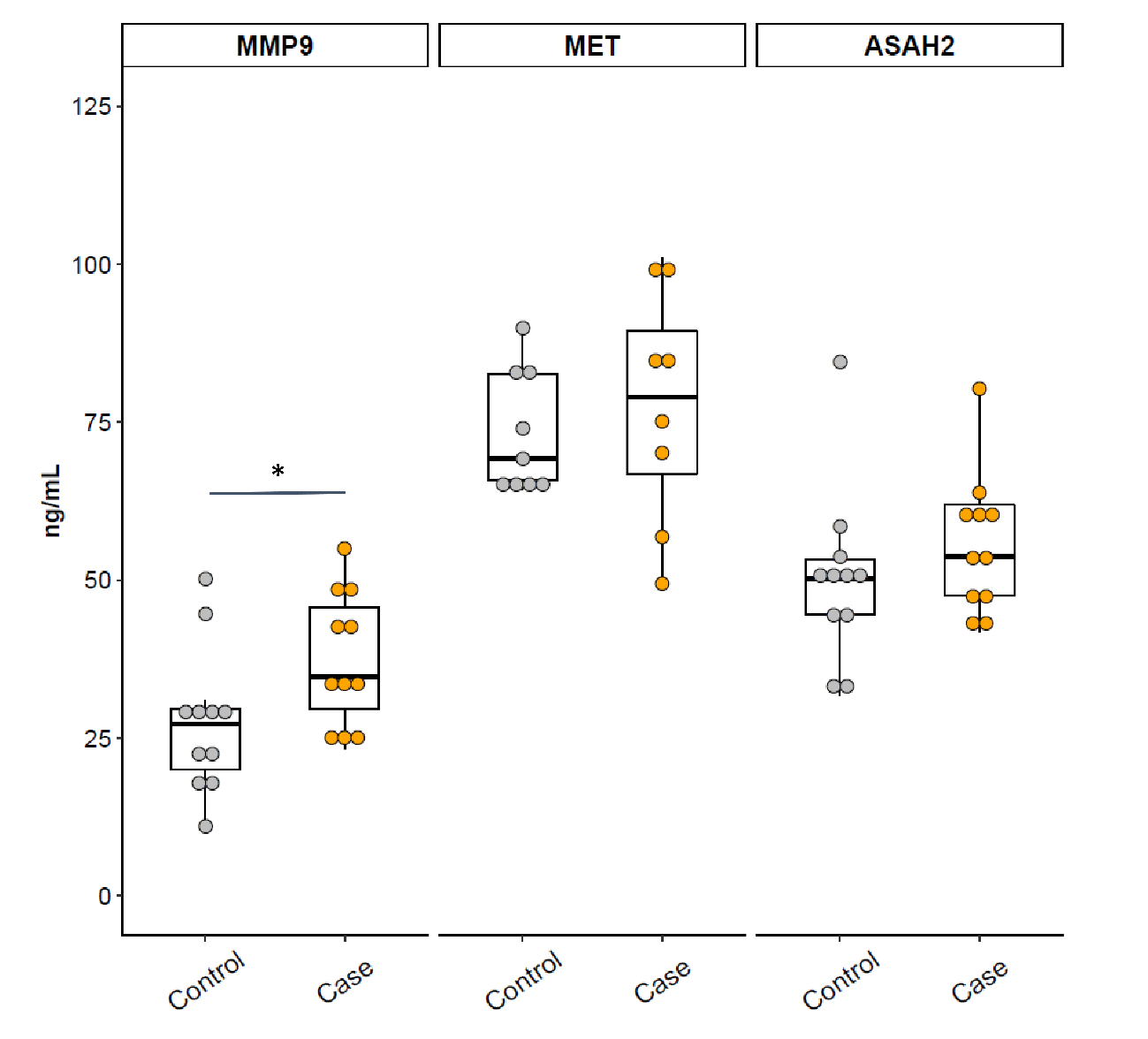


**Supplementary Figure 1. Boxplots representing baseline biomarker concentrations of the replication phase in subjects who additionally belonged to the discovery study (N=22).**

Y axis represents baseline concentrations in biomarkers tested in the replication phase. X axis represents the group (cases versus controls). Each panel represents one protein (MMP9, MET and ASAH2). Results can be compared with those of figure 2-B.

Missing data: 3 out of 11 pairs missed baseline MET concentration in the replication phase.

*: *p*-value<0.05.


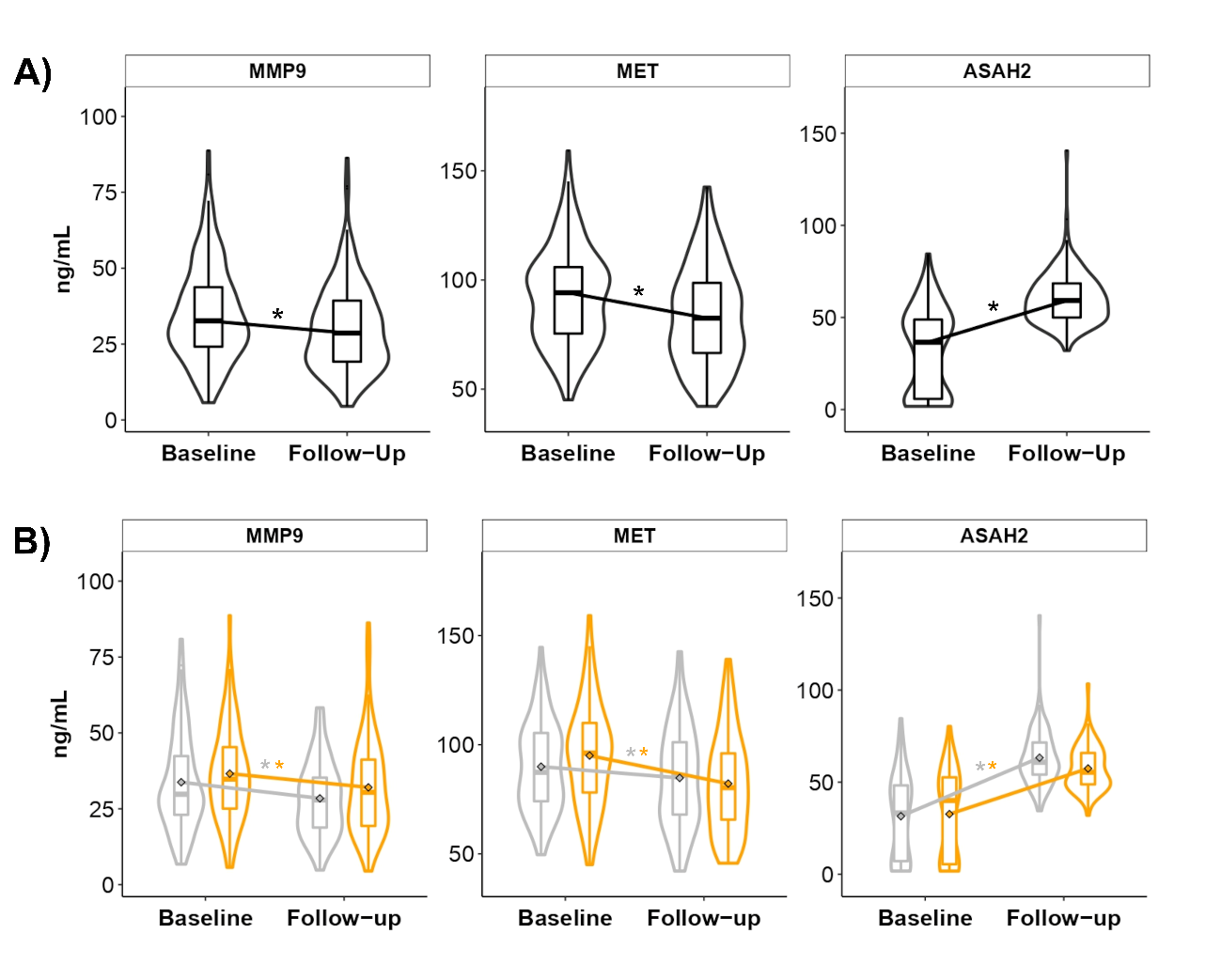


**Supplementary figure 2.** **Boxplots showing the temporal profile of biomarkers from the replication phase.**

Violin plots show the distribution (empirical density function) of those biomarkers chosen in the replication phase, while boxplots display the median and the inter-quartile range (N=160). Lines represent the changes in the concentration (Y axis) from baseline to follow-up visit (X axis). Row A shows the change in MMP9, MET and ASAH2 in the whole sample, while in row B these results are stratified by group (controls in grey, and cases in orange). In row B, the small diamonds represent the mean of each group at each time.

Missing data: 13 samples were missing considering MET (7 were baseline samples and 6 were follow-up samples) and 12 for ASAH2 (all baseline samples).

*: Repeated measures *p*-value<0.01.

Key: ASAH2, neutral ceramidase; MET, hepatocyte growth factor receptor; MMP9, matrix metalloproteinase-9; RFU, relative fluorescence units.


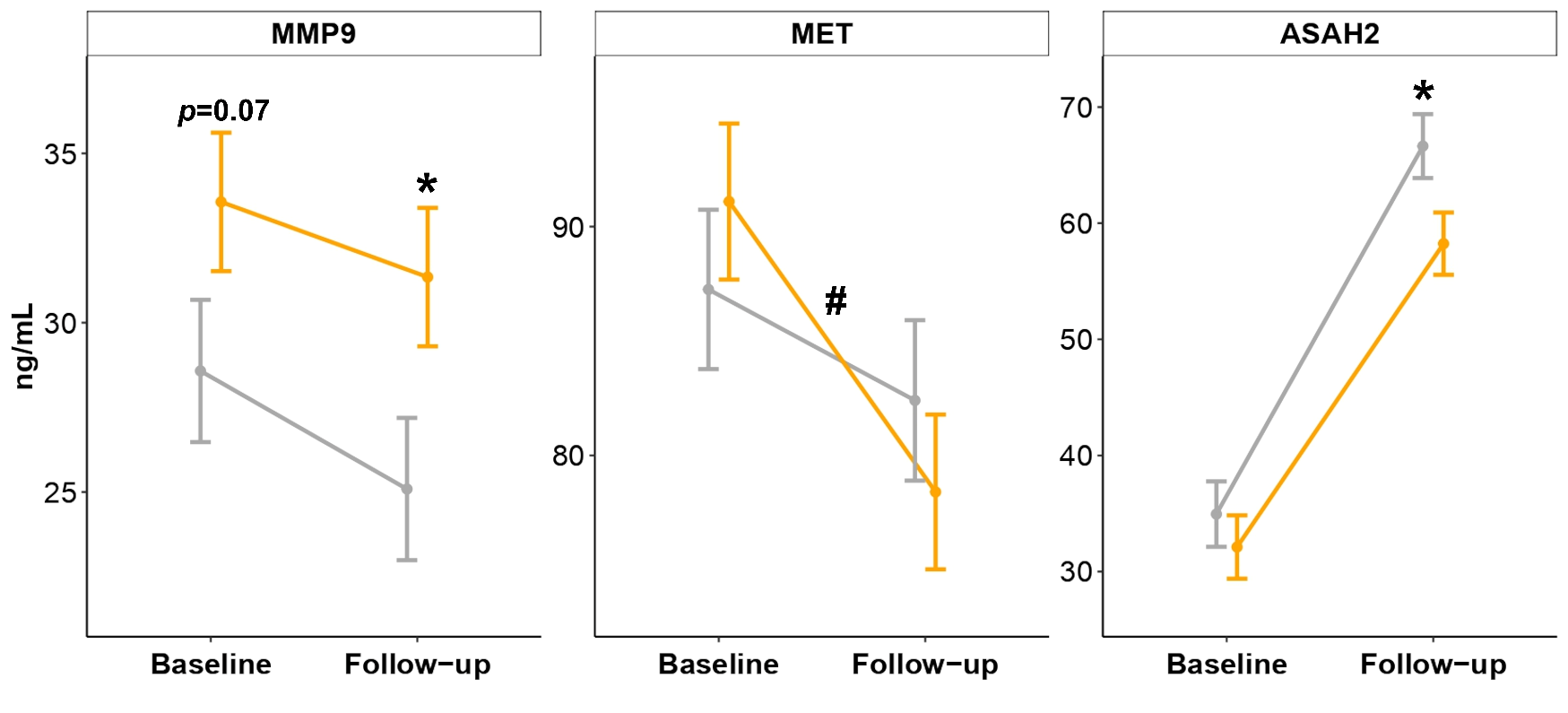


**Supplementary Figure 3**. **Sensitivity analysis representing biomarker concentrations in pairs of subjects having equal WMH burden (N=110).** Lines represent adjusted means from baseline to the follow-up visit in cases (orange) and controls (grey). Error bars represent the standard error of the adjusted mean. These adjusted means have been extracted from mixed models corrected for: baseline age, sex, visit, change in systolic blood pressure and baseline WMH.

After excluding pairs of subjects showing differences in baseline WMH, we obtained equivalent results as in the whole sample (see Figure 3).

*: *p*-value<0.05 between groups at the baseline visit or at the follow-up visit.

#: group and visit interaction *p*-value<0.05. This interaction term represents the difference in the rate of changes between groups in biomarker concentration.

| **Supplementary table 1. Characteristics of the discovery phase sample by cases and controls** | | | |
| --- | --- | --- | --- |
|  | **Controls (n = 12)** | **Cases (n = 12)** | ***p*-value** |
| Baseline Age, years | 64.0 (62.3-67.8) | 63.5 (62.0-67.0) | 0.909 |
| Sex | 6 (50.0) | 6 (50.0) | 1 |
| Time between visits, years | 4.0 (3.8;4.2) | 3.9 (3.8;4.7) | 0.453 |
| **Vascular risk factors** | | | |
| Diabetes mellitus | 3 (25.0) | 0 (0) | 0.220 |
| Active Smoker | 0 (0) | 3 (25.0) | 0.220 |
| SBP, mmHg | 145.3 (11.3) | 138.3 (15.2) | 0.208 |
| **ΔSBP, mmHg** | **-3.4 (11.5)** | **11.1 (16.8)** | **0.024** |
| DBP, mmHg | 78.2 (10.6) | 77.3 (9.6) | 0.834 |
| ΔDBP, mmHg | -3.5 (7.4) | -0.5 (7.9) | 0.361 |
| Cholesterol, mg/dL | 214.3 (42.3) | 222.7 (44.5) | 0.639 |
| ΔCholesterol, mg/dL | 8.9 (44.3) | -14.2 (31.4) | 0.156 |
| HDL Cholesterol, mg/dL | 49.6 (14.9) | 51.5 (13.0) | 0.742 |
| ΔHDL Cholesterol, mg/dL | 2.8 (0.0;5.3) | 4.4 (2.0;10.2) | 0.441 |
| **Baseline Cerebral Small Vessel Disease** | | | |
| Extensive PVH | 3 (25.0) | 3 (25.0) | 1 |
| Extensive DWMH | 2 (16.7) | 4 (33.3) | 0.640 |
| Silen brain infarct | 0 (0) | 3 (25.0) | 0.220 |

Values represent mean (SD), median (interquartile range) or number (%). Bolded variables are those which show significant differences between cases and controls (P-value<0.05).

Key: DBP, diastolic blood pressure; DWMH, deep white matter hyperintensities; HDL, high density lipoprotein; PVH, periventricular hyperintensities; SBP, systolic blood pressure.

| **Supplementary table 2. Proteins differentially expressed in the discovery study** | | | | | |
| --- | --- | --- | --- | --- | --- |
| **Gene name** | **Uniprot Code** | **logFC** | ***t-*statistic** | ***p-*value** | ***q-*value** |
| MET | P08581 | -0.211 | -2.867 | 0.0084 | 0.9366 |
| ASAH2 | Q9NR71 | -0.382 | -2.858 | 0.0085 | 0.9366 |
| IDS | P22304 | -0.395 | -2.838 | 0.0090 | 0.9366 |
| MMP9 | P14780 | 0.482 | 2.796 | 0.0099 | 0.9366 |
| CD200R1 | Q8TD46 | -0.268 | -2.756 | 0.0108 | 0.9366 |
| IL5RA | Q01344 | -0.263 | -2.740 | 0.0113 | 0.9366 |
| F2 | P00734 | -0.324 | -2.707 | 0.0122 | 0.9366 |
| ADGRE2 | Q9UHX3 | -0.279 | -2.695 | 0.0125 | 0.9366 |
| SEMA3E | O15041 | -0.497 | -2.673 | 0.0131 | 0.9366 |
| GPC3 | P51654 | -0.326 | -2.598 | 0.0156 | 0.9366 |
| CGA TSHB | P01215 P01222 | -0.579 | -2.516 | 0.0188 | 0.9366 |
| SEMA6A | Q9H2E6 | -0.440 | -2.491 | 0.0198 | 0.9366 |
| CLEC7A | Q9BXN2 | 0.278 | 2.482 | 0.0203 | 0.9366 |
| CD109 | Q6YHK3 | -0.390 | -2.477 | 0.0205 | 0.9366 |
| PGK1 | P00558 | -0.258 | -2.435 | 0.0225 | 0.9366 |
| DCN | P07585 | -0.211 | -2.395 | 0.0245 | 0.9366 |
| CADM1 | Q9BY67 | -0.309 | -2.373 | 0.0258 | 0.9366 |
| A2M | P01023 | -0.255 | -2.370 | 0.0260 | 0.9366 |
| COLEC12 | Q5KU26 | -0.392 | -2.347 | 0.0273 | 0.9366 |
| MPO | P05164 | 0.352 | 2.321 | 0.0289 | 0.9366 |
| BRF1 | Q92994 | 0.227 | 2.302 | 0.0301 | 0.9366 |
| CST5 | P28325 | -0.619 | -2.262 | 0.0328 | 0.9366 |
| BMPER | Q8N8U9 | 0.203 | 2.261 | 0.0329 | 0.9366 |
| NR1D1 | P20393 | 0.281 | 2.252 | 0.0335 | 0.9366 |
| CCL26 | Q9Y258 | 0.202 | 2.244 | 0.0341 | 0.9366 |
| C4A C4B | P0C0L4, P0C0L5 | 0.119 | 2.243 | 0.0342 | 0.9366 |
| ITGAV ITGB5 | P06756, P18084 | 0.367 | 2.240 | 0.0344 | 0.9366 |
| NTRK3 | Q16288 | -0.253 | -2.232 | 0.0349 | 0.9366 |
| PROC | P04070 | 0.163 | 2.222 | 0.0357 | 0.9366 |
| FTH1 FTL | P02794 P02792 | -0.569 | -2.218 | 0.0361 | 0.9366 |
| IL1R1 | P14778 | -0.537 | -2.185 | 0.0386 | 0.9366 |
| SLITRK5 | O94991 | -0.427 | -2.157 | 0.0410 | 0.9366 |
| BOC | Q9BWV1 | -0.426 | -2.147 | 0.0418 | 0.9366 |
| SGTA | O43765 | -0.520 | -2.146 | 0.0419 | 0.9366 |
| EDA | Q92838 | -0.295 | -2.145 | 0.0420 | 0.9366 |
| POMC | P01189 | 0.175 | 2.141 | 0.0423 | 0.9366 |
| PLXNB2 | O15031 | -0.133 | -2.106 | 0.0456 | 0.9366 |
| SPINT2 | O43291 | -0.241 | -2.096 | 0.0465 | 0.9366 |
| GDNF | P39905 | 0.133 | 2.094 | 0.0468 | 0.9366 |
| C5 | P01031 | 0.275 | 2.086 | 0.0475 | 0.9366 |
| LAG3 | P18627 | -0.317 | -2.071 | 0.0490 | 0.9366 |

Proteins differentially expressed in the discovery study. LogFC (log fold change) and *t*­-statistic correspond to mean_cases_ – mean_controls_ contrast. Adjusted *q*-values have been calculated with false discovery rate correction.

| **Supplementary table 3: Enriched Gene Sets in the discovery phase.** | | | | |
| --- | --- | --- | --- | --- |
| **ID** | **Name** | **Size** | ***p*-value** | **FDR *q-*value** |
| REAC:R-HSA-6803157 | Antimicrobial peptides | 97 | <0.0001 | <0.0001 |
| GO:0019199 | Transmembrane receptor protein kinase activity | 82 | <0.0001 | 0.0222 |
| GO:0004714 | Transmembrane receptor protein tyrosine kinase activity | 64 | <0.0001 | 0.0278 |
| GO:0004888 | Transmembrane signaling receptor activity | 1244 | <0.0001 | 0.1439 |
| REAC:R-HSA-1442490 | Collagen degradation | 64 | 0.0029 | 0.1528 |
| GO:0004252 | Serine-type endopeptidase activity | 247 | <0.0001 | 0.1735 |
| GO:1901343 | Negative regulation of vasculature development | 35 | <0.0001 | 0.1752 |
| GO:0140110 | Transcription regulator activity | 2068 | 0.0201 | 0.1796 |
| REAC:R-HSA-6798695 | Neutrophil degranulation | 477 | <0.0001 | 0.1813 |
| GO:0008134 | Transcription factor binding | 654 | 0.0300 | 0.1815 |
| GO:0019838 | Growth factor binding | 139 | 0.0091 | 0.1863 |
| GO:1990837 | Sequence-specific double-stranded DNA binding | 848 | 0.0377 | 0.1869 |
| GO:0017171 | Serine hydrolase activity | 274 | <0.0001 | 0.1873 |
| GO:0003712 | Transcription coregulator activity | 561 | 0.0334 | 0.1898 |
| GO:0001012 | RNA polymerase II regulatory region DNA binding | 758 | 0.0388 | 0.1899 |
| GO:0000977 | RNA polymerase II regulatory region sequence-specific DNA binding | 751 | 0.0606 | 0.1911 |
| REAC:R-HSA-3781865 | Diseases of glycosylation | 138 | 0.0028 | 0.1952 |
| GO:0043565 | Sequence-specific DNA binding | 1131 | 0.0206 | 0.199 |
| GO:0001540 | Amyloid-beta binding | 75 | 0.0168 | 0.2019 |
| GO:0044212 | Transcription regulatory region DNA binding | 919 | 0.0242 | 0.2046 |
| REAC:R-HSA-1630316 | Glycosaminoglycan metabolism | 124 | 0.0015 | 0.2087 |
| GO:0000978 | RNA polymerase II proximal promoter sequence-specific DNA binding | 513 | 0.0718 | 0.2192 |
| GO:0000987 | Proximal promoter sequence-specific DNA binding | 527 | 0.0901 | 0.2208 |
| GO:0004713 | Protein tyrosine kinase activity | 182 | 0.0024 | 0.2212 |
| GO:0003690 | Double-stranded DNA binding | 938 | 0.0335 | 0.2269 |
| GO:0001067 | Regulatory region nucleic acid binding | 921 | 0.0205 | 0.2328 |
| GO:0016525 | Negative regulation of angiogenesis | 32 | <0.0001 | 0.2347 |
| GO:0038023 | Signaling receptor activity | 1462 | 0.0023 | 0.2443 |

Enriched pathways (*q*-value<0.25) obtained from the GSEA analysis in the discovery phase (N=24, panel background=1305 proteins). Values represent the size of the complete pathway, and the *p-*values and *q*-values of the overlap.

| **Supplementary table 4: Enriched gene sets involving the biomarkers candidates** | |
| --- | --- |
| **MMP9** | **MET** |
| Collagen Degradation (REAC) | Signaling receptor activity (GO) |
| Neutrophil degranulation (REAC) | Transmembrane signaling receptor activity (GO) |
| Serine hydrolase activity (GO) | Transmembrane receptor protein kinase activity (GO) |
| Serine-type endopeptidase activity (GO) | Transmembrane receptor protein tyrosine kinase activity (GO) |
|  | Protein tyrosine kinase activity (GO) |

Enriched pathways including the biomarker candidates. Parentheses indicate whether the pathway was obtained from the gene-ontologies (GO) or reactome (REAC) databases. None of the 28 enriched pathways included ASAH2.

| **Supplementary Table 5. Characteristics of the replication phase in subjects showing no differences in baseline WMH** | | |
| --- | --- | --- |
|  | **Controls (N=55)** | **Cases (N=55)** |
| Baseline Age, years | 64 (61;67) | 64 (62;66) |
| Sex | 31 (56.4) | 31 (56.4) |
| Time between visits, years | 4.0 (3.8;4.1) | 4.0 (3.8;4.3) |
| Vascular risk factors | | |
| Diabetes mellitus | 9 (16.4) | 8 (14.6) |
| Active Smoker | 5 (9.1) | 6 (10.9) |
| SBP, mmHg | 144.5 (16.2) | 139.9 (18.0) |
| **ΔSBP, mmHg** | **3.1 (13.8)** | **9.8 (18.0)** |
| DBP, mmHg | 78.3 (9.4) | 79.1 (9.5) |
| ΔDBP, mmHg | -3.5 (8.8) | -1.0 (10.2) |
| Cholesterol, mg/dL | 217.1 (43.3) | 211.6 (43.9) |
| ΔCholesterol, mg/dL | -3.7 (40.5) | -7.5 (40.5) |
| HDL Cholesterol, mg/dL | 46.1 (39.1;60.2) | 47.5 (41.9;57.6) |
| ΔHDL Cholesterol, mg/dL | 3.9 (-0.3;9.0) | 3.4 (-1.6;7.8) |
| Baseline Cerebral Small Vessel Disease | | |
| Extensive PVH | 9 (16.4) | 8 (14.6) |
| Extensive DWMH | 3 (5.5) | 8 (14.6) |
| Silent brain infarct | 7 (12.7) | 15 (27.3) |

Values represent mean (SD), median (interquartile range) or number (%). Bolded variables are those which show significant differences between cases and controls (P-value<0.05).

Key: DBP, diastolic blood pressure; DWMH, deep white matter hyperintensities; HDL, high density lipoprotein; PVH, periventricular hyperintensities; SBP, systolic blood pressure.
